# Zinc diamyldithiocarbamate: MAK Value Documentation – Translation of the German version from 2023

**DOI:** 10.34865/mb1533718kske10_4or

**Published:** 2025-12-22

**Authors:** Andrea Hartwig, MAK Commission

**Affiliations:** 1 Institute of Applied Biosciences. Department of Food Chemistry and Toxicology. Karlsruhe Institute of Technology (KIT) Adenauerring 20a, Building 50.41 76131 Karlsruhe Germany; 2 Permanent Senate Commission for the Investigation of Health Hazards of Chemical Compounds in the Work Area. Deutsche Forschungsgemeinschaft, Kennedyallee 40, 53175 Bonn, Germany. Further information: Permanent Senate Commission for the Investigation of Health Hazards of Chemical Compounds in the Work Area | DFG

**Keywords:** zinc diamyldithiocarbamate, toxicity, MAK value, developmental toxicity, germ cell mutagenicity, carcinogenicity, sensitization, peak limitation, Luft, air

## Abstract

The German Senate Commission for the Investigation of Health Hazards of Chemical Compounds in the Work Area (MAK Commission) has evaluated the data for zinc diamyldithiocarbamate [15337-18-5] to derive an occupational exposure limit value (maximum concentration at the workplace, MAK value) considering all toxicological end points. Relevant studies were identified from a literature search and also unpublished study reports were used. Zinc diamyldithiocarbamate does not lead to specific systemic toxicity, presumably due to low levels of uptake. Irritating effects induced by zinc diamyldithiocarbamate are only minimal, thus an oral study can be used to derive a maximum concentration at the workplace (MAK value). A combined study investigating reproductive toxicity and repeated dose toxicity that was carried out with rats according to OECD Test Guideline 422 determined a NOAEL of 85 mg/kg body weight and day after oral administration. This dose has been scaled to a MAK value of 10 mg/m^3^ I (inhalable fraction). Exposure to the inhalable fraction results in gastrointestinal exposure via mucociliary clearance, reducing peak concentrations. Therefore, the substance has been assigned to Peak Limitation Category II with an excursion factor of 8. Similar to the findings after exposure to white mineral oil, inhalation of the aerosol of the poorly water-soluble zinc diamyldithiocarbamate may lead to lung overload, inflammatory reactions and microgranulomas. To prevent these overload effects, a MAK value of 5 mg/m^3^ has been derived for the respirable fraction in analogy to white mineral oil and Peak Limitation Category II with an excursion factor of 4 has been set. There are no prenatal teratogenicity studies with zinc diamyldithiocarbamate. Therefore, zinc diamyldithiocarbamate has been assigned to Pregnancy Risk Group D. The substance is not genotoxic in vitro; there are no in vivo data. No carcinogenicity studies have been carried out. There is no evidence that zinc diamyldithiocarbamate has sensitizing potential. Skin contact is not expected to contribute significantly to systemic toxicity.

**Table:** 

**MAK value (2022)**	**5 mg/m^3^ R (respirable fraction)** **10 mg/m^3^ I (inhalable fraction)**
**Peak limitation (2022)**	**R fraction:** **Category II, excursion factor 4** **I fraction:** **Category II, excursion factor 8**
	
**Absorption through the skin**	**–**
**Sensitization**	**–**
**Carcinogenicity**	**–**
**Prenatal toxicity (2022)**	**Pregnancy Risk Group D**
**Germ cell mutagenicity**	**–**
	
**BAT value**	**–**
	
Synonyms	zinc bis(dipentyldithiocarbamate)
Chemical name (IUPAC)	zinc *N*,*N*-dipentylcarbamodithioate
CAS number	15337-18-5
Structural formula	((C_5_H_11_)_2_N–CS–S)_2_Zn
Molecular formula	C_22_H_44_N_2_S_4_Zn
Molar mass	530.23 g/mol
Melting point	9 °C; OECD Test Guideline 102 (ECHA 2020)
Boiling point at 1032 hPa	320 °C; OECD Test Guideline 103 (ECHA 2020)
Density at 20 °C	1.14 g/cm^3^; OECD Test Guideline 109 (ECHA 2020)
Vapour pressure at 25 °C	6.3 × 10^–13^ hPa; OECD Test Guideline 104 (ECHA 2020)
log K_OW_ at 30 °C	> 9.4; OECD Test Guideline 117 (ECHA 2020)
Solubility at 20 °C	0.086 mg/l water; OECD Test Guideline 105 (ECHA 2020)
	
Hydrolytic stability	no data
Stability	no data
Production	no data
Purity	96.6% (ECHA 2020)
Impurities	no data
Uses	antioxidant, metal deactivator, copper corrosion inhibitor, colour stabilizer, antiwear agent used in engine and industrial oils and in greases; accelerator for natural and synthetic rubbers (Parchem fine & specialty chemicals 2022)
Concentrations used	no data

The documentation is based primarily on the registration data publicly available under REACH (ECHA 2020). All the studies available for zinc diamyldithiocarbamate were carried out recently according to the latest OECD test guidelines. Cited unpublished toxicological studies from companies have been made available to the Commission.

## 1 Toxic Effects and Mode of Action

The low water solubility of 0.086 mg/l, the log K_OW_ of > 9.4 and the high molar mass of zinc diamyldithiocarbamate suggest a low oral and dermal uptake. Accordingly, no effects were reported in an acute toxicity study with rats given zinc diamyldithiocarbamate in an oral dose of 2000 mg/kg body weight. In a 14-day range-finding study for a combined study of the toxic effects of repeated oral doses and a screening test for reproductive toxicity in Crl:CD(SD) rats, severe toxicity was observed at dose levels of 500 and 1000 mg/kg body weight and day within the first 3 days of treatment. The study was terminated prematurely as a result. On day 2 of treatment, irregular respiration and ruffled fur were transient effects observed in 1 of the 3 males at 250 mg/kg body weight and day. The non-specific, but marked toxicity occurred again in the main study and led to the sacrifice of 1 female at 250 mg/kg body weight. Another animal was in a poor general state of health, and the body weight gains were reduced in all female animals.

Zinc diamyldithiocarbamate did not cause skin irritation in an in vitro test with reconstructed human epidermis. The substance caused minimal conjunctival irritation in the rabbit eye, which was reversible within 72 hours. Zinc diamyldithiocarbamate was not sensitizing in a local lymph node assay (LLNA) in female CBA/Ca mice. There are no studies available for sensitizing effects on the airways. Zinc diamyldithiocarbamate was not mutagenic in bacteria either in the presence or in the absence of metabolic activation and did not induce micronuclei in human lymphocytes or genotoxic effects in the TK^+/–^ test in L5178Y mouse lymphoma cells. Studies of zinc diamyldithiocarbamate that investigated genotoxicity in vivo or carcinogenicity are not available.

## 2 Mechanism of Action

There are no data available.

## 3 Toxicokinetics and Metabolism

There are no studies available.

The low water solubility of 0.086 mg/l, the log K_OW_ of > 9.4 and the high molar mass of zinc diamyldithiocarbamate suggest a low oral and dermal uptake (ECHA 2020). Mathematical models cannot be used to calculate absorption through the skin because the substance has a log K_OW_ that is greater than 6.

## 4 Effects in Humans

There are no studies available.

However, there is information about allergenic effects caused by structurally similar compounds. Dithiocarbamates can be oxidized to thiurams; as potent contact allergens, they may form conjugates with serum albumin or other peptides or proteins with free thiol groups (Chipinda et al. 2008; Hartwig and MAK Commission 2016).

While the 2 homologous structural analogues zinc di**methyl**dithiocarbamate (ziram) and zinc di**ethyl**dithiocarbamate were found to cause pronounced sensitization, the sensitizing effects caused by zinc di**butyl**dithiocarbamate in humans seem to be milder (see for example Aalto-Korte and Pesonen 2016). Lipophilicity and molecular size increase with the chain length. As a result, structural analogues with longer chains may permeate to a lesser degree, leading to a lower sensitizing potential. Thus, when patients with occupational contact dermatitis and suspected allergy to gloves were patch tested, 3.3% (n = 1987) reacted to zinc di**ethyl**dithiocarbamate, but only 0.4% (n = 1377) reacted to zinc di**butyl**dithiocarbamate. However, zinc di**butyl**dithiocarbamate was used less frequently in the past (Geier et al. 2003).

## 5 Animal Experiments and in vitro Studies

### 5.1 Acute toxicity

#### 5.1.1 Inhalation

There are no data available.

#### 5.1.2 Oral administration

In a study of acute toxicity that was carried out in 2018 according to OECD Test Guideline 420, 1 female Wistar rat (RccHan™:WIST) was initially given a gavage dose of zinc diamyldithiocarbamate (purity: 96.6%) in dimethyl sulfoxide of 2000 mg/kg body weight. As no signs of toxicity were observed, another 4 animals were given zinc diamyldithiocarbamate in an oral dose of 2000 mg/kg body weight. The animals were observed for 14 days and then underwent gross-pathological examination, which did not yield any unusual findings. Therefore, the LD_50_ was higher than 2000 mg/kg body weight (ECHA 2020).

In an earlier study from 1978, groups of 5 male Sherman Wistar rats were given zinc diamyldithiocarbamate in doses of 1000, 2000, 4000, 8000 or 16 000 mg/kg body weight. The animals were observed for 14 days. The highest dose was lethal for 3 of the 5 animals within 12 to 18 hours. Two hours after administration of the substance, the animals of this dose group first became lethargic and then comatose before dying. The gross-pathological examination did not yield any unusual findings. The LD_50_ was 14 900 mg/kg body weight (ECHA 2020).

#### 5.1.3 Dermal application

There are no data available.

### 5.2 Subacute, subchronic and chronic toxicity

#### 5.2.1 Inhalation

There are no data available.

#### 5.2.2 Oral administration

A combined study of the toxic effects of repeated oral doses and of reproductive toxicity is available, which was carried out in 2018 according to OECD Test Guideline 422 (Table 1).

**Tab. 1:** Toxicity after repeated oral administration of zinc diamyldithiocarbamate in Crl:CD(SD) rats (Envigo CRS Limited 2018 b)

**Dose [mg/kg body weight and day]**	**Findings**
28 and above	♂: haematocrit values ↓ (not dose-dependent), ♀: erythrocyte count ↑ (dose dependency uncertain);
**85**	**NOAEL**
85 and above	♂: MCV, bilirubin ↓ (dose dependency uncertain);
250	♂: marginal increase in relative weights of Cowper’s gland (increase attributed to 2 animals); ♀: 1/10 (day 12), activity ↓, ruffled fur, irregular gait, tremor, hunched posture, sacrificed in extremis, mild mineralization of the renal papillae, minimal unilateral tubular basophilia of the kidneys, minimal ulceration in the glandular stomach, minimal atrophy of the uterus; 1/10: poor general state, ruffled fur from days 12 to 15, closed eyes some of the time, staining of the snout; minimal decrease in feed consumption from days 1 to 15 and statistically significant decrease during gestation and lactation; reduced body weights on days 8 and 15 (not statistically significant), decrease in body weight gains from days 1 to 20 of gestation; statistically significant decrease in relative weights of the heart, cervix and oviducts

MCV: mean corpuscular erythrocyte volume

The doses tested in the main study were selected based on the results from a 14-day range-finding study, which was carried out with gavage doses of 0, 100, 250, 500 and 1000 mg/kg body weight and day. At 500 and 1000 mg/kg body weight and day, severe toxicity was observed within the first 3 days. Therefore, the treatment of these dose groups was terminated prematurely. Gross-pathological findings were detected mainly in the stomach in addition to clinical signs such as reduced or increased activity, irritated behaviour, ruffled fur, hunched posture, irregular gait, irregular or increased respiration, tremor and closed eyes some of the time. On day 2, 1 of 3 males of the group given 250 mg/kg body weight transiently exhibited irregular respiration and ruffled fur.

In the main study, groups of 10 male and 10 female Crl:CD(SD) rats were given 96.6% zinc diamyldithiocarbamate dissolved in arachis oil once daily in gavage doses of 0, 28, 85 or 250 mg/kg body weight and day. The males were treated for at least 5 weeks beginning 2 weeks before mating, while the females were treated from 2 weeks before mating, throughout mating and gestation, up to day 13 of lactation. The females and their offspring were sacrificed and examined on day 14 of lactation. On day 12 of treatment, 1 female of the high dose group was sacrificed because the animal was in a poor general state of health with symptoms including reduced activity, ruffled fur, irregular gait, tremor and hunched posture. The histopathological examination of the animal revealed mild mineralization of the renal papillae, minimal unilateral tubular basophilia of the kidneys, minimal ulceration of the glandular stomach and minimal atrophy of the uterus. From days 12 to 15 of treatment, ruffled fur, closed eyes some of the time and staining of the snout were observed in another female of this group. In addition, compared with the values recorded for the control group, this female did not gain body weight from days 1 to 8 and its body weight was reduced between days 8 and 15. Chromodacryorrhoea was observed in 1 female of the group that received a dose of 28 mg/kg body weight.

The body weight gains of the females in the middle and high dose groups were reduced from days 8 to 15, but the decreases were not statistically significant. During gestation, the body weight gains of the group that received 250 mg/kg body weight were reduced markedly and with statistical significance compared with the values determined in the control animals. The final body weights were thus reduced with statistical significance in this group. The feed consumption of the females that received 250 mg/kg body weight was reduced slightly from days 1 to 15 of treatment and with statistical significance during gestation and lactation.

At the end of treatment, the mean corpuscular erythrocyte volume was reduced slightly, but with statistical significance, in the males of the middle and high dose groups, and the haematocrit values were slightly reduced at the low dose and above, but without dose dependency (see Table 2). These effects were marginal and within the historical control data of the laboratory. Therefore, the authors of the study do not consider them to be related to treatment. At the end of the study, the number of erythrocytes was increased with statistical significance in the females at the low dose and above, and the bilirubin values were reduced with statistical significance in the females of the middle and high dose groups. These effects were likewise marginal and within the historical control data of the laboratory. Therefore, the authors of the study and the Commission do not consider them to be related to treatment.

**Tab. 2:** Effects on the blood count of Crl:CD(SD) rats (n = 5; means (± standard deviation)) after repeated oral administration of zinc diamyldithiocarbamate (Envigo CRS Limited 2018 b)

**Dose [mg/kg body weight and day]**	**♂: haematocrit [l/l]**	**♂: MCV [fl]**	**♀: erythrocytes [× 10^12^/l]**	**♀: bilirubin [µmol/l]**
0	0.417 (0.0066)	53.8 (1.17)	6.27 (0.200)	1 (0.0)
28	0.395 (0.0217)*	53.0 (0.92)	6.62 (0.242)*	1 (0.4)
85	0.380 (0.0093)*	52.0 (0.95)*	6.61 (0.164)*	0 (0.4)**
250	0.400 (0.0154)*	52.0 (0.79)*	6.75 (0.220)**	0 (0.4)**

* p ≤ 0.05;

** p ≤ 0.01 (pairwise t-test)

fl: femtolitre; MCV: mean corpuscular erythrocyte volume

The T4 (thyroxin) values of the males were unchanged compared with those of the untreated control animals.

In the males of the high dose group, the mean relative weights of Cowper’s gland were increased with statistical significance after treatment for 5 weeks. This increase was attributed to 2 individual animals and was not regarded as related to treatment because no other findings were observed. In the females of the high dose group, the relative weights of the heart, cervix and oviducts were reduced with statistical significance at the end of treatment on day 14 of lactation. These effects were not considered to be related to treatment because they were marginal and no histopathological correlates were observed. The gross-pathological and histopathological examinations of the treated animals did not yield any unusual findings. On the basis of the data, the authors of the study regarded the dose of 250 mg/kg body weight and day to be the NOAEL (no observed adverse effect level) (Envigo CRS Limited 2018 b).

The non-specific, but clearly toxic findings, particularly those observed in the females at 250 mg/kg body weight and day (1 of 10 females sacrificed in extremis and another sacrificed in a poor general state of health as well as reduced body weight gains), are consistent with the findings observed in the 14-day range-finding study at 500 mg/kg body weight and day. The range-finding study was discontinued at this dose level because of severe toxicity. Therefore, the dose of 85 mg/kg body weight and day is considered to be the NOAEL of this study. The effects on the blood count that were reported at 28 mg/kg body weight and day and above are not regarded as adverse by the Commission because there is no consistency between the findings or between the sexes.

#### 5.2.3 Dermal application

There are no data available.

### 5.3 Local effects on skin and mucous membranes

#### 5.3.1 Skin

In an in vitro test carried out in 2018 according to OECD Test Guideline 439 with reconstructed human epidermis (EpiSkin^TM^), 3 test samples were treated for 15 minutes with 10 µl zinc diamyldithiocarbamate (purity: 96.6%), corresponding to 26.3 µl/cm^2^. At the end of treatment, the tissues were rinsed and kept for another 42 hours without treatment. A subsequent MTT test (3-(4,5-dimethylthiazol-2-yl)-2,5-diphenyltetrazolium bromide) showed the survival of the cells treated with the test substance to be 120.7% in comparison with the control tissue, which was set as 100%. The positive control sample (sodium lauryl sulfate 5% w/v) verified the functioning of the test system. The standard deviation of 19.3% calculated for the 3 tests carried out with zinc diamyldithiocarbamate was slightly above the limit of the acceptance criteria for the test (≤ 18%). However, the above-mentioned deviation did not affect the integrity or validity of the study because the results were clearly negative (survival was > 100%). On the basis of this test, zinc diamyldithiocarbamate is considered not to cause skin irritation (ECHA 2020).

#### 5.3.2 Eyes

A study of eye irritation caused by zinc diamyldithiocarbamate (purity: 96.6%) was carried out in 2 male New Zealand White rabbits according to OECD Test Guideline 405. For this purpose, 0.1 ml of the test substance was instilled into one eye of each of the 2 animals and irritation was recorded after 1 hour and after 24, 48 and 72 hours. During treatment, the animals were given a subcutaneous injection of an analgesic. Effects on the iris or cornea were not found. Moderate conjunctival irritation was observed after 1 hour; this was minimal after 24 and 48 hours and reversible after 72 hours. The irritation scores for both animals were 9, 5, 2 and 0 of a maximum of 80 after 1 hour and after 24, 48 and 72 hours, respectively. The body weight of 1 of the 2 animals was reduced at the end of the study. The substance is regarded as a weak irritant. However, the irritation scores do not fulfil the criteria for classifying the substance as irritating to the eyes according to the Globally Harmonized System of Classification and Labelling of Chemicals (Envigo CRS Limited 2018 a).

### 5.4 Allergenic effects

#### 5.4.1 Sensitizing effects on the skin

The sensitizing potential of zinc diamyldithiocarbamate (purity: 96.6%) was investigated in an LLNA carried out in female CBA/Ca mice according to OECD Test Guideline 429. The 25% and 50% formulations in acetone/olive oil (4:1 v/v) and the undiluted test substance yielded stimulation indices of 1.6, 1.65 and 2.36, respectively. Therefore, the test substance is not considered to be sensitizing (ECHA 2020; Envigo CRS Limited 2018 c).

In an LLNA carried out with the structural analogue zinc di**butyl**dithiocarbamate, an EC3 value could not be calculated because a stimulation index of 3.0 was not reached with test concentrations up to 20%. If these results are compared with those of LLNAs carried out with zinc di**methyl**dithiocarbamate and zinc di**ethyl**dithiocarbamate, the calculated EC3 values reveal a decreasing sensitizing potential: zinc diethyldithiocarbamate > zinc dimethyldithiocarbamate >> zinc dibutyldithiocarbamate (De Jong et al. 2002).

Considered together with the negative results of the LLNA for zinc diamyldithiocarbamate, it is assumed that the sensitizing potential of dithiocarbamates containing zinc decreases with an increase in the alkyl chain length. Zinc diamyldithiocarbamate, therefore, has no (or only a very weak) sensitizing potential.

#### 5.4.2 Sensitizing effects on the airways

There are no data available.

### 5.5 Reproductive and developmental toxicity

#### 5.5.1 Fertility

In a combined study of reproductive toxicity and of the toxic effects of repeated oral doses carried out according to OECD Test Guideline 422, zinc diamyldithiocarbamate was given to groups of 10 male and 10 female Crl:CD(SD) rats in daily gavage doses of 0, 28, 85 or 250 mg/kg body weight and day. Details of the test procedure and the effects on the parental animals are described in Section 5.2.2. The fertility parameters precoital interval, fertility, mating performance, gestation period and gestation index were not affected by the treatment. Therefore, the NOAEL for impairment of fertility was 250 mg/kg body weight and day. Examination of the testes, seminiferous tubules and sperm did not yield any findings. As described in Section 5.2.2, the mean relative weights of Cowper’s gland were increased with statistical significance in the males at 250 mg/kg body weight and day. However, this increase resulted from the findings in just 2 animals and was therefore regarded as incidental. On day 12 of gestation, 1 female that was given a dose of 250 mg/kg body weight and day was sacrificed in extremis, and the histopathological examination revealed minimal atrophy of the uterus. The females and their offspring were sacrificed and examined on day 14 of lactation. In the females, the combined relative weights of the uterus, cervix and oviducts were reduced with statistical significance. These effects were regarded as incidental because they were marginal and no histopathological correlates were observed (Envigo CRS Limited 2018 b).

#### 5.5.2 Developmental toxicity

Developmental toxicity studies are not available.

In the above-mentioned study carried out according to OECD Test Guideline 422, zinc diamyldithiocarbamate was given to groups of 10 male and 10 female Crl:CD(SD) rats in daily gavage doses of 0, 28, 85 or 250 mg/kg body weight and day. Details of the test procedure and the effects on the parental animals are described in Section 5.2.2. The length of gestation remained unchanged and pre-implantation and post-implantation losses or dead foetuses did not occur. On postnatal day 1, the mean body weights of the offspring of the animals that received the high dose of 250 mg/kg body weight and day were slightly reduced compared with those of the controls. This finding was not considered to be related to the treatment because it was not statistically significant. The number of live foetuses, the sex ratio, litter size and weights and the postnatal survival of the offspring were not affected by treatment with zinc diamyldithiocarbamate. Likewise, no unusual findings were obtained for the anogenital distance of the male and female offspring and the number of nipples in the male offspring. There was no evidence of external malformations (Envigo CRS Limited 2018 b). Therefore, the NOAEL for perinatal toxicity was 250 mg/kg body weight and day. At this dose level, marked toxicity was observed in the parental animals (see Section 5.2.2).

In a study carried out according to OECD Test Guideline 422, the offspring are examined only externally without skeletal or visceral examinations. For this reason, teratogenicity was not fully evaluated.

### 5.6 Genotoxicity

#### 5.6.1 In vitro

In a Salmonella mutagenicity test carried out in 2018 according to OECD Test Guideline 471, the strains Salmonella typhimurium TA98, TA100, TA1535 and TA1537 and Escherichia coli WP2 uvr A (pKM 101) were incubated with zinc diamyldithiocarbamate (purity: 96.6%, dissolved in dimethyl sulfoxide) in concentrations of 0, 5, 15, 50, 150, 500, 1500 or 5000 µg/plate both in the presence and in the absence of a metabolic activation system. Two independent tests were carried out: the first, a plate incorporation test and the second, a preincubation test. In the first test, no cytotoxicity was observed up to the highest concentration; however, the test substance precipitated at 5000 µg/plate both in the presence and in the absence of a metabolic activation system. In the second test, cytotoxicity was detected in the strain TA1537 at 50 µg/plate in the presence of metabolic activation. This was not regarded as a treatment-induced effect because it was neither related to the dose nor was it observed in the other cultures. Zinc diamyldithiocarbamate was not mutagenic in any of the test cultures. The positive controls verified the functioning of the test system (ECHA 2020).

Zinc diamyldithiocarbamate (purity: 96.6%, dissolved in acetone) was tested in a micronucleus test with human lymphocytes that was carried out in 2018 according to OECD Test Guideline 487 both in the presence and in the absence of a metabolic activation system. The lymphocytes were isolated from the pooled blood samples of 2 healthy, non-smoking volunteers. In the pretest, the cells were incubated with concentrations of 0, 3.91, 7.81, 15.63, 31.25, 62.5, 125, 250, 500, 1000 or 2000 μg/ml. In the first main test, they were initially incubated with concentrations up to 20 μg/ml because visible precipitates had formed in the pretest at concentrations of 15.63 μg/ml and above after incubation for 3 hours. As neither precipitates nor cytotoxicity were found in the main test at concentrations of 0.25, 2.5, 5, 10, 15 or 20 μg/ml either in the presence or in the absence of a metabolic activation system, the test was carried out with higher concentrations. The new concentrations were 0.1, 10, 15, 20, 22.5, 25, 27.5, 30 and 35 μg/ml. After incubation of the lymphocytes with zinc diamyldithiocarbamate for 3 hours with and without metabolic activation, the test substance precipitated at 30 μg/ml with concurrent findings of cytotoxicity in 36.1% and 30.7% of the cells, respectively (lower CBPI (cytokinesis-block proliferation index) compared with the values determined in the controls). Therefore, the concentrations of 0.1, 20 and 30 μg/ml were used for the evaluation of the micronucleus test. The number of binuclear cells with micronuclei was not increased compared with the control number. The 20-hour treatment of lymphocytes with concentrations of 0.0025, 0.025, 0.25, 2.5, 5, 10, 15 or 20 μg/ml in the presence of a metabolic activation system led to cytotoxicity in 53.2% of the cells at 15 μg/ml compared with the findings in the controls. Therefore, the concentrations of 0.25, 5 and 15 μg/ml were used for the evaluation of the micronucleus test. Zinc diamyldithiocarbamate did not induce micronuclei in human lymphocytes in any of the test cultures. The positive controls verified the functioning of the test system (ECHA 2020).

A TK^+/–^ test in L5178Y mouse lymphoma cells carried out in 2018 according to OECD Test Guideline 490 investigated the genotoxic effects of zinc diamyldithiocarbamate (purity: 96.6%, dissolved in acetone) both in the presence and in the absence of a metabolic activation system. In a pretest, the relative total growth (RTG) was reduced at concentrations of 3.91 to 2000 µg/ml after 3-hour incubation with and without metabolic activation. Therefore, concentrations of 0.5, 5, 15, 30, 40, 50 and 60 µg/ml were chosen for the main test. On the basis of the pretest, concentrations of 0.05, 0.5, 5, 10, 15, 20, 25 and 30 µg/ml were selected for 24-hour exposure without the addition of a metabolic activation system. Zinc diamyldithiocarbamate did not induce genotoxic effects in any of the test cultures. After treatment for 3 hours, both in the presence and in the absence of a metabolic activation system, the RTG values at 40 and 30 µg/ml were 19% and 16%, respectively. Therefore, these were the highest concentrations that were included in the evaluation. After 24-hour treatment, the RTG value was 19% at 10 µg/ml, the highest concentration included in the evaluation. The positive controls verified the functioning of the test system (ECHA 2020).

#### 5.6.2 In vivo

There are no data available.

### 5.7 Carcinogenicity

There are no data available.

## 6 Manifesto (MAK value/classification)

Zinc diamyldithiocarbamate does not induce specific systemic toxicity, presumably because it is not absorbed readily.

**MAK value. **The metal dithiocarbamate ziram causes severe lung toxicity (MAK value: 0.01 mg/m^3^; Hartwig and MAK Commission 2016). Ziram is corrosive to the eyes, whereas zinc diamyldithiocarbamate causes only minimal eye irritation (Envigo CRS Limited 2018 a) and no skin irritation is observed (ECHA 2020). Therefore, zinc diamyldithiocarbamate is not expected to cause marked toxic effects on the respiratory tract. Oral administration does not induce local effects on the stomach or intestines, as were observed after the administration of ziram; likewise, no lung toxicity has been described. A MAK value can be derived from the oral study because zinc diamyldithiocarbamate causes only minimal irritation.

**Inhalable fraction**: A combined study of the toxic effects of repeated oral doses and of reproductive toxicity carried out in rats according to OECD Test Guideline 422 reported a NOAEL of 85 mg/kg body weight and day (see Section 5.2.2). There are no quantitative data available for oral absorption of the substance.

The following toxicokinetic data are used to extrapolate the oral NOAEL of 85 mg/kg body weight and day to a concentration in workplace air: the daily exposure of the animals in comparison with exposure for 5 days a week at the workplace (7:5), the corresponding species-specific correction value for the rat (1:4), the body weight (70 kg) and the respiratory volume (10 m^3^) of the person. Absorption after oral and inhalation exposure have not been included in the calculation because of the poor solubility of the substance, which leads to the removal of the inhalable fraction that is deposited in the upper respiratory tract by swallowing. For this reason, the amounts taken up after inhalation and oral exposure are equivalent. The corresponding concentration in the air is thus calculated to be 208 mg/m^3^.

Taking into consideration that this value was derived from a NOAEL from an animal study (1:2) and that the effects may increase with time (1:6), this results in a concentration of 17 mg/m^3^. By applying the preferred value approach, a MAK value of 10 mg/m^3^ I has been derived for the inhalable fraction.

**Respirable fraction**: As zinc diamyldithiocarbamate is not readily soluble in water and has a high log K_OW_, absorption of the respirable fraction is expected to be low. However, an inhalation study that investigated this end point is not available. Zinc diamyldithiocarbamate is a viscous liquid. The risk of accumulation is low because this substance is deposited as an aerosol with a high log K_OW_ on a surfactant film that consists of 90% lipids. Mixing is thus likely to occur. The surfactant has a half-life of less than 24 hours (Schulz 2017), and it is assumed that the xenobiotic substance is degraded. If large amounts are deposited, the physical function of the surfactant and thus the physical dynamics of the alveoli may be impaired.

The properties in the lungs are therefore similar to those of mineral oil (water solubility: < 0.1 mg/l depending on the carbon number) (Hartwig and MAK Commission 2019). A MAK value of 5 mg/m^3^ for the respirable fraction was derived from inhalation studies for the effects induced by mineral oil on the lungs. This value would provide protection both against the systemic toxicity of zinc diamyldithiocarbamate (see above) and against any potential toxicity in the lungs. Therefore, in analogy to that for mineral oil, a MAK value of 5 mg/m^3^ has been established for the respirable fraction of zinc diamyldithiocarbamate, as was set for another dithiocarbamate, methylene bis(dibutyldithiocarbamate) (Hartwig and MAK Commission 2023).

**Peak limitation. Inhalable fraction**: After inhalation of the inhalable fraction, the substance is deposited in the respiratory tract. Systemic exposure occurs after removal of the substance by swallowing following mucociliary clearance. Therefore, the substance has been classified in Peak Limitation Category II. Concentration peaks are levelled out because the substance is distributed in the respiratory tract and this clearance has its own half-life. Therefore, the same excursion factor of 8 has been established as for methylene bis(dibutyldithiocarbamate) (Hartwig and MAK Commission 2023).

**Respirable fraction**: The respirable fraction has been assigned to Peak Limitation Category II with an excursion factor of 4 in analogy to mineral oil (Hartwig and MAK Commission 2019).

**Prenatal toxicity. **Developmental toxicity studies are not available.

In a study carried out according to OECD Test Guideline 422 with Crl:CD(SD) rats given gavage doses of zinc diamyldithiocarbamate, effects on the general state of health and reduced body weight gains were observed in the females at 85 mg/kg body weight and day. However, no substance-induced effects were found in the males up to the highest dose tested. Therefore, the NOAEL for perinatal toxicity was the high dose of 250 mg/kg body weight and day (Envigo CRS Limited 2018 b).

A study carried out according to OECD Test Guideline 422 does not include a full evaluation of developmental toxicity. Therefore, zinc diamyldithiocarbamate has been classified in Pregnancy Risk Group D.

**Carcinogenicity and germ cell mutagenicity. **Zinc diamyldithiocarbamate was not genotoxic in a mutagenicity test in Salmonella typhimurium, in a micronucleus test in human lymphocytes or in a TK**^+/–^** mutation test with L5178Y mouse lymphoma cells; all 3 tests were carried out according to OECD test guidelines. Studies of genotoxicity in vivo or carcinogenicity are not available; however, these kinds of effects are not assumed because of the structure of the substance. Therefore, the currently available data do not give cause to classify zinc diamyldithiocarbamate in any of the categories for carcinogens or germ cell mutagens.

**Absorption through the skin. **There are no studies available for absorption through the skin. The models for calculating absorption through the skin cannot be used because of the high log K_OW_. An in vitro test that investigated skin irritation did not reveal cytotoxic effects and thus found no evidence of absorption through the skin. As absorption through the skin is expected to be quite low because of the low solubility of the substance in water and its high log K_OW_, zinc diamyldithiocarbamate has not been designated with an “H” (for substances which can be absorbed through the skin in toxicologically relevant amounts).

**Sensitization. **There are no findings available from humans and no positive results from animal studies. Therefore, zinc diamyldithiocarbamate has not been designated with “Sh” (for substances which cause sensitization of the skin). No data are available for sensitization of the respiratory tract. Zinc diamyldithiocarbamate has thus not been designated with “Sa” (for substances which cause sensitization of the airways).

## References

[ref_DND6RX6W] Aalto-Korte Kristiina, Pesonen Maria (2016). Patterns of simultaneous patch test reactions to thiurams and dithiocarbamates in 164 patients. Contact Dermatitis.

[ref_8IAQHQQ2] Chipinda Itai, Hettick Justin M., Simoyi Reuben H., Siegel Paul D. (2008). Zinc diethyldithiocarbamate allergenicity: potential haptenation mechanisms.. Contact Dermatitis.

[ref_JHR6I8M2] De Jong Wim H., Van Och François M. M., Den Hartog Jager Constance F., Spiekstra Sander W., Slob W., Vandebriel Rob J., Van Loveren Henk (2002). Ranking of allergenic potency of rubber chemicals in a modified local lymph node assay.. Toxicol Sci.

[ref_D48RIQCV] (2020). Zinc bis(dipentyldithiocarbamate) (CAS Number 15337-18-5). Registration dossier. Joint submission, first publication 15 May 2018, last modification 10 May 2020.

[ref_8ZG5JG54] Envigo CRS Limited (2018). Zinc bis (dipentyldithiocarbamate) (oil free): acute eye irritation in the rabbit.

[ref_P7UUXWVB] Envigo CRS Limited (2018). Zinc bis (dipentyldithiocarbamate) (oil free): combined repeated dose toxicity study with reproductive/developmental toxicity screening in the rat by oral administration.

[ref_Y9BZDI8G] Envigo CRS Limited (2018). Zinc bis (dipentyldithiocarbamate) (oil free): local lymph node assay in the mouse – individual method.

[ref_MND8ZYKY] Geier Johannes, Lessmann Holger, Uter Wolfgang, Schnuch Axel (2003). Occupational rubber glove allergy: results of the Information Network of Departments of Dermatology (IVDK), 1995–2001.. Contact Dermatitis.

[ref_2BJRL5YB] Hartwig A, MAK Commission (2016). Ziram. MAK Value Documentation, 2015. MAK Collect Occup Health Saf.

[ref_54BVFZYK] Hartwig A., MAK Commission (2019). Mineral oils (petroleum), severely refined. MAK Value Documentation, 2018. MAK Collect Occup Health Saf.

[ref_QB965NZ6] Hartwig Andrea, MAK Commission (2023). Methylene bis(dibutyldithiocarbamate). MAK Value Documentation – Translation of the German version from 2020. MAK Collect Occup Health Saf.

[ref_MD5N8KFC] Parchem fine & specialty chemicals (2022). Zinc diamyldithiocarbamate, product description.

[ref_P4WJJBEM] Schulz F., Greim H. (2017). Das Toxikologiebuch. Grundlagen, Verfahren, Bewertung.

